# Simple surgical punctal occlusion with high frequency radiowave electrosurgery

**DOI:** 10.1186/s12886-023-02798-4

**Published:** 2023-02-02

**Authors:** Jeong Woo Park, Jisang Han, Wan Kyu Choi, Jaemin Kim, Chul Young Choi

**Affiliations:** 1 Department of Ophthalmology, Kangbuk Samsung Hospital, Sungkyunkwan University School of Medicine, 29 Saemunan-Ro, Jongno-Gu, Seoul, 03181 South Korea; 2 Department of Medicine, Graduate School, Kyung Hee University, Seoul, South Korea

**Keywords:** Sjögren’s syndrome, Dry eye, Punctal occlusion high frequency radiowave

## Abstract

**Background:**

To introduce and evaluate the efficacy of a simple punctal occlusion technique for dry eye patients.

**Methods:**

Medical records of 79 eyes from 40 patients refractory to common dry eye conservative treatment who underwent multiple high-frequency radio-wave electro-punctal occlusion were retrospectively reviewed. Pre- and post-procedural ocular surface indices (Schirmer test, tear break-up time (TBUT), and corneal staining grade (Oxford scheme)) and subjective symptom scores (including frequency of artificial tear use, interval between procedures, and total repeat time) were analyzed.

**Results:**

Average Schirmer test result was significantly (*P* < 0.05) improved from 4.10 ± 1.39 mm to 8.14 ± 3.13 mm at 6 weeks after the procedure (*n* = 79). A total of 32 eyes from 16 patients underwent repeated procedure with a mean interval of 8.00 ± 4.86 months, while 24 patients had a single procedure. Twenty-five of 30 patients showed improvement for subjective symptom scores. No complications related to the procedure were observed.

**Conclusions:**

A simple, less-invasive punctal occlusion technique using a fine-needle tip with high-frequency radio-wave could significantly relieve subjective symptoms and improve ocular surface indices of patients with aqueous deficient dry eye without causing a major complication. This procedure may play a considerable role in treating dry eye refractory to common practices.

**Supplementary Information:**

The online version contains supplementary material available at 10.1186/s12886-023-02798-4.

## Background

Dry eye disease is a multifactorial disease that can show various symptoms due to changes in tear film homeostasis. According to its pathophysiology, dry eye disease can be divided into evaporative dry eye (EDE) caused by Meibomian gland dysfunction, blepharitis, blinking, SCL wear, vitamin A deficiency, topical drug preservatives, ocular surface diseases and Accutane use and aqueous deficient dry eye (ADDE) caused by decreased tear secretion including Sjögren’s syndrome (SS) lacrimal deficiency, lacrimal duct obstruction and systemic drug use [1, 2].

Besides supplementing the lack of tears simply with artificial tears for treating dry eye syndrome, various medical treatment options such as lipid supplementation and autologous serum depending on the cause of the disease are also available. Interventional treatment method with punctal occlusion such as punctal plug can help the ocular surface to keep the natural tear film for a longer duration. It is recommended for ADDE [3].

Punctal occlusion can be broadly classified into non-surgical and surgical methods. Punctal plug is a typical example of a non-surgical method. The plug can be used temporarily or permanently depending on the material and model. It is easy to manipulate. Several studies have demonstrated its effectiveness in improving symptoms and ocular surface indices in patients with various dry eye syndromes [4–6]. However, it may irritate the eye or become unable to serve its purpose due to self-extrusion [7]. In addition, inflammation and granuloma formation related to punctal plug may occur [8, 9]. Furthermore, infections including dacryocystitis might develop [10]. Surgical methods include punctal cauterization, conjunctival flap, punctal plug suturing, and extirpation of canaliculus. Coagulating punctum and canaliculi using thermal cautery has been used in clinical trials. Its usefulness has been proven in several studies [11–13]. However, this method should be carefully considered because it can cause irreversible tissue damage.

Devices using radiofrequency wave have been used in ophthalmology and other medical fields as they are convenient and safe. In addition, they allow precise manipulation. High-frequency wave has less heat dispersion than conventional cautery. Thus, there might be less damage to surrounding tissues with a shallow depth of the burn, resulting in less scarring, adhesion, and changes in appearance after the procedure. In addition, it has the advantage of a quick recovery [14, 15].

The purpose of this study was to investigate the clinical usefulness of punctal coagulation using high-frequency radio-wave with relatively low temperatures (7–80°) for ADDE patients refractory to general management. 

## Methods

### Subjects

Medical records of 79 eyes from 40 dry eye disease (DED) patients with tear deficiency who were refractory to the maximum tolerable medical treatment using eyedrops including 1% prednisolone eyedrops and cyclosporine eyedrops (0.05% or 0.1%) in ophthalmology outpatient clinic of Kangbuk Samsung Hospital between January 2017 and December 2021 were retrospectively analyzed. Inclusion criteria were clinically diagnosed ADDE with or without SS by Schirmer test with anesthesia < 5.0 mm in 2 sequential exams with more than 1 month interval. We also excluded patients with EDE components like meibomian gland dysfunction, abnormal eyelid contour, abnormal blinking, contact lens wearing, and ocular surface diseases like allergic conjunctivitis, vitamin A deficiency. Diagnosis with Sjögren syndrome is done by rheumatologist according to 2016 American College of Rheumatology/European League Against Rheumatism classification criteria with anti-SSA/Ro antibody test and labial gland biopsy result.

### Surgical procedures

Under usual drape, topical anesthesia was administered with 0.5% proparacaine (Alcaine®, Novartis, NJ, USA). Radiofrequency electrode tip (OcuRF**®,** Ilooda, Korea) was inserted into the lower lacrimal punctum of patient. Radio-wave released from electric unit (0.6 ~ 0.8 Watt, 2 MHz, Acutron™, Ilooda, Korea) to coagulate punctal mucosa 1–2 mm away from the punctal opening for 1–2 s. The same procedure was repeated 2–3 times per punctum to coagulate broader area of punctal lumen. A mucosal burn was created from the deeper canaliculi (about 1 ~ 2 mm away from punctal opening) to the direction of external punctal opening [see Additional file 1]. Caution was given not to coagulate the external surface too much which can cause surface scar of the punctum. Just after the coagulation, cold wet compression was applied to the punctal opening.

**Fig. 1:**
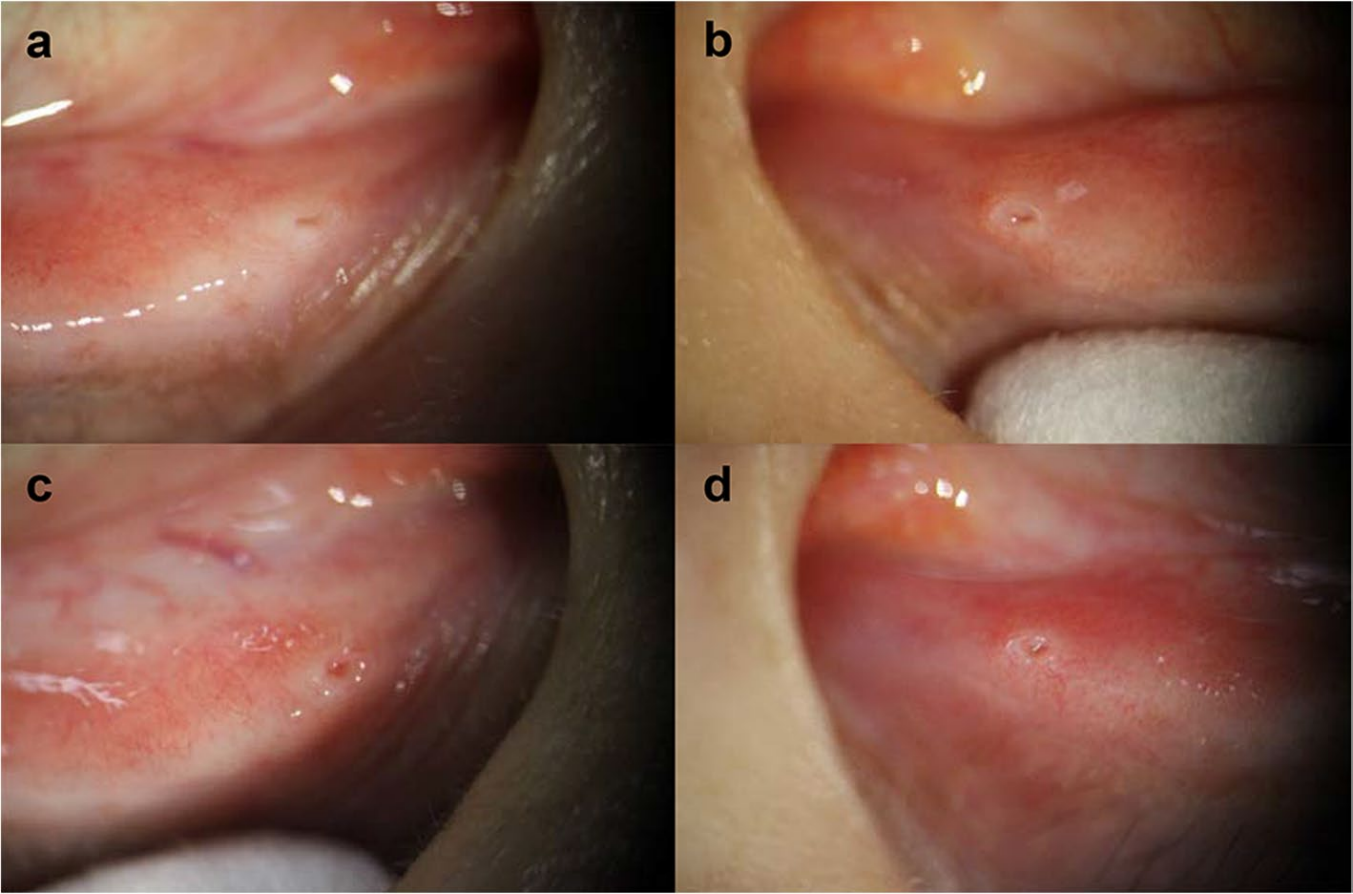
Clinical photographs of each lower lacrimal punctum of the same patient (#3) before (**a**, **b**) and after (**c**, **d**) the first procedure. Grossly notable closure or stenosis is not seen around the patient’s punctum

For two severe Sjögren's syndrome dry eye (SSDE) patients showing limited improvement with both lower punctal coagulation, four punctal occlusions in both eyes were performed. Limitation of improvement was determined by worsening corneal staining score and symptom score, Schirmer's test, and limiting improvement in BUT. Figure 1 shows pictures of lower punctum before the procedure and at one day after the procedure. No significant morphological change in punctal shape was noted under slit lamp examination after the procedure.

### Outcome measures

In this study, the routine follow-up schedule after RF punctal occlusion was 6 weeks after the procedure, and additional treatment schedule was determined according to the patient's condition. Cornea was stained with 5 μL of 2% fluorescein solution. After blinking, tear break-up time (TBUT) was examined three times by the same examiner using a slit-lamp microscope under cobalt blue filter/yellow filter in the slit lamp. The average value was recorded. Corneal and conjunctival staining grade was examined after TBUT according to the Oxford scheme for ocular surface grading [16].

Schirmer test was done with Schirmer strip (ColorBar, Eagle vision) which was placed in the outer third of lower fornix. After 5 min, the value was measured in millimeters.

We did not perform a standardized questionnaire such as the Ocular Surface Disease Index (OSDI) on all patients in an outpatient setting. Therefore, based on the records of the medical chart, the patient's symptoms were classified and scaled from 0 to 5 points. Based on severity of pain, visual discomfort, and lubricants-free intervals, severe symptoms were scored as 0 point and complete improvement was scored as 5 points.

### Total repeated number of procedure & interval between procedures

For patients who had multiple procedures during the follow-up period, the interval between procedures was analyzed. For these patients, changes in data of ocular surface indices were evaluated once before and after the first procedure. Patients who were lost to follow-up for more than 12 months were excluded.

### Statistical analysis

All data were analyzed with Statistical Package for the Social Science (SPSS) 24.0 (IBM Corp., Armonk, NY, USA). Paired t-test or the Wilcoxon signed rank test was performed for every outcome measure both before and after the procedure. Independent t-test or the Wilcoxon rank sum test was performed after normality test to compare changes of ocular surface indices between SSDE group & non-Sjögren’s syndrome dry eye (NSDE) group. *P* < 0.05 was regarded as statistically significant for each analysis.

## Results

Records of 79 eyes from a total of 40 patients were retrospectively analyzed. The average age of the patient group was 60.8 years. This study included 9 (22.5%) men and 31 (77.5%) women. The number of patients with Sjögren’s syndrome dry eye (SSDE) was 9 (22.5%). All of them were women. Thirty-one (77.5%) patients with NSDE included patients on estrogen hormone replacement therapy, anti-depressive agents, and chemotherapy (Table 1).

**Table 1:** Preoperative characteristics of patients

	**Total (*n* = 40)** **Mean ± SD (%)**	**SSDE (*n* = 9)** **Mean ± SD (%)**	**NSDE (*n* = 31)** **Mean ± SD (%)**
Age(years)	60.8 ± 12.8	56.2 ± 16.4	62.1 ± 11.5
SEX			
MALE	9 (22.5)	0	9 (29.0)
FEMALE	31 (77.5)	9 (100.0)	22 (71.0)
Systemic diseases			
Hypertension	12 (30.0)	2 (22.2)	10 (32.6)
Diabetes	10 (25.0)	3 (33.3)	7 (22.6)
Medication history			
Hormone therapy	2 (5.0)	-	2 (6.5)
Anti-depressant	2 (5.0)	-	2 (6.5)
Chemotherapy	3 (7.5)		3 (9.8)
Ophthalmologic history			
Laser refractive surgery		1 (11.1)	
Prostaglandin analogue use	3 (7.5)		3 (9.7)
IOP	14.3 ± 2.50	14.8 ± 2.86	14.1 ± 2.53
BCVA (LogMAR)	0.12 ± 0.13	0.15 ± 0.18	0.11 ± 0.13

Data are presented as mean ± standard deviation or number (%)

*IOP* Intraocular pressure, *SSDE* Sjögren’s syndrome dry eye, *NSDE* Non-Sjögren’s syndrome dry eye, *BCVA* Best corrected visual acuity

Tear break-up time showed significant improvement in all groups (pre-procedure vs. post-procedure: 2.99 ± 1.76 s vs. 5.70 ± 2.34 s, *P* < 0.01, *n* = 24, Table 2). Both SSDE and NSDE groups showed significant improvement in tear break-up time (Tables 3 and 4). The amount of improvement between the two groups was not significantly different (Table 5).

**Table 2:** Changes of ocular surface indices & symptom score before and after the procedure (both groups had significant improvement of these indices & symptom score without showing statistically significant difference between the two groups)

	**Pre**	**Post**	**Difference**	*P* value^*^
TBUT(sec)	2.99 ± 1.76	5.70 ± 2.34	+ 2.51 ± 2.63 (*n* = 24)	< 0.01
Corneal staining(grade)	2.44 ± 0.71	1.20 ± 1.01	-1.24 ± 1.05 (*n* = 34)	< 0.01
Schirmer test(mm)	4.10 ± 1.39	8.14 ± 3.13	+ 4.04 ± 3.39 (*n* = 79)	< 0.01
Symptom score(score)	2.39 ± 0.61	3.64 ± 0.82	+ 1.25 ± 0.59 (*n* = 30)	< 0.01

*TBUT* Tear breakup time

^*^P values were from paired t-test

**Table 3:** Ocular surface indices in Sjögren’s syndrome dry eye patients

	**n**	**-**	**Pre**	**Post**	**Difference**	*P* value^*^
TBUT(sec)	6	Median (IQR)	2 (1.93, 3.05)	4.9 (4.08, 5)	1.65 (0.18, 2.83)	0.049
Corneal staining(grade)	10	Median (IQR)	2.5 (2, 3)	1 (1, 2)	-1.5 (-2, -1)	0.012
Schirmer test(mm)	18	Median (IQR)	3 (3, 5)	6.5 (5, 10)	3.5 (2.25, 5)	0.001
Symptom score(score)	12	Median (IQR)	2.5 (2, 3)	4 (4, 5)	-2 (-2, -2)	0.002

*TBUT* Tear breakup time, *Pre* before the procedure, *Post* post the procedure

^*^ *P* values were from the Wilcoxon signed rank test

**Table 4:** Ocular Surface Indices in non-Sjögren’s syndrome dry eye patients

	**Pre**	**Post**	**Difference**	*P* value^*^
TBUT (sec)	3.15 ± 1.90	6.07 ± 2.42	+ 2.83 ± 2.87 (*n* = 18)	< 0.01
Corneal staining (grade)	2.41 ± 0.77	1.19 ± 1.06	-1.21 ± 1.14 (*n* = 24)	< 0.01
Schirmer test (mm)	4.21 ± 1.44	8.44 ± 3.25	+ 4.23 ± 3.54 (*n* = 61)	< 0.01
Symptom score (score)	2.37 ± 0.63	3.51 ± 0.79	+ 1.14 ± 0.55 (*n* = 18)	< 0.01

*TBUT* Tear break-up time

^*^P values were from paired t-test

**Table 5:** Comparison of ocular surface indices change between the two groups

	-	**SSDE**	**NSDE**	***P* value**
TBUT(sec)	Median (IQR)	1.65 (0.18, 2.83)	2 (0, 6)	0.477^*^
Corneal staining(grade)	Mean ± SD	-2.7 ± 1.34	-2.46 ± 1.86	0.672^†^
Schirmer test(mm)	Median (IQR)	3.5 (2.25, 5)	4 (1, 6)	0.573^*^

*NSDE* Non-Sjögren’s syndrome dry eye, *SSDE* Sjögren’s syndrome dry eye, *TBUT* Tear break-up time

^*^P values were from the Wilcoxon rank sum test

^†^*P* values were from independent t-test

After the procedure, corneal staining grade showed significant decrease in all the groups (pre-procedure vs. post-procedure: 2.44 ± 0.71 vs. 1.20 ± 1.01, *P* < 0.01, *n* = 34, Table 2). Both SSDE and NSDE groups showed significant improvement in corneal staining grade, but no significant difference was seen between the groups (Tables 3, 4 and 5).

In Schirmer test, both groups showed significant increase (4.04 ± 3.34), with a mean pre-operative value of 4.10 ± 1.39 and a post-operative value of 8.14 ± 3.13 (*P* < 0.01, *n* = 79, Table 2). Similar results were obtained for individual groups separately (Tables 3 and 4).

Of 40 patients, 24 underwent a single procedure while the remaining 16 underwent repeated procedure (at least twice). Among patients with SS, 9 (100%) patients received the procedure more than twice. The proportion of SS was significantly higher in the group that received repeated procedures than that in the group undergoing the procedure only once (*P* < 0.05). The mean interval between the first and second procedure was 8.00 ± 4.86 months (range, 2 to 28 months). One patient underwent the procedure up to 7 times within 3 years. Of 24 patients who received a single procedure, 13 were lost to follow up. In all procedures, no patient complained of continuous pain, sense of irritation, or epiphora during the follow-up period.

## Discussion

According to the DEWS report (2007), dry eye is classified into aqueous-deficient type and evaporating type. However, many patients tend to have both aspects. ADDE is composed of Sjögren's syndrome dry eye (SSDE) and non-Sjögren's syndrome dry eye (NSDE). Besides SS, ADDE can occur by various causes such as systemic drug, Graft-versus-host reaction or disease (GVHD), refractive surgery, chemical burn, cicatricial conjunctival disease, and so on [17].

For the management of aqueous deficient dry eye, various types of ocular lubricants are considered as primary treatments. Those who are refractory to these treatments are recommended to use punctal plug, moisture chamber, and immunomodulatory drugs as secondary treatments [3].

Plugs of various materials and shapes are being used in punctal occlusion method. Many studies have shown that the use of punctal plugs for patients with dry eye syndrome can significantly improve symptoms and ocular surface index. While punctal plug is easy to perform, complications such as self-extrusion, granuloma formation, and infection including dacryocystitis may also occur. According to a study by Ming-Cheng et al., when silicone punctal plug was used in dry eye patients who were refractory to the maximal tolerable medical treatment, it significantly improved ocular surface indices such as corneal erosion and Schirmer test [5]. A study performed by Balaram et al. also showed similar results. However, punctal plugs were spontaneously lost due to self-extrusion in both studies [18]. Besides, plug associated complications such as granuloma formation and bacterial biofilm formation have been reported [3]. Thus, close care should be taken during the treatment.

Thermal punctal occlusion, one of surgical options, directly coagulates the punctum or canaliculi using thermal cautery, diathermy, and argon laser. These invasive processes may cause side effects including pain, epiphora, and lower lid deformation depending on the degree of the invasiveness [3, 19]. According to a study by Emii Ohba et al., permanent punctal occlusion by commercially available thermal cautery can improve symptoms as well as objective value and index in a patient with recurrent punctal plug extrusion. Also, the re-canalization rate was low [12]. However, permanent punctal occlusion should be performed carefully because there is an irreversible risk when side effects including epiphora occur. Ricardo et al. have studied partial punctal occlusion, a method for reducing punctum diameter using thermal cautery. They found that this method could significantly improve symptoms and ocular surface index in patients with Sjogren syndrome [13]. Nevertheless, cauterizing the punctal opening may induce lid abnormalities such as ectropion and entropion, eventually causing side effects and irritating the ocular surface by coagulating the punctum.

In this study, we induced thermal shrinkage to the canalicular mucosa with a simple punctal occlusion technique using radiofrequency energy with electrode tips mounted on 2 MHz electric device. This results in mucosal burn induced by heat from tissue resistance and high-frequency radio-waves in deep canaliculi. Punctal opening can be relatively spared from limited heat dispersion to the surface skin. This RF treatment has been proven to be effective in conjunctivochalasis at first in our previous studies, and high frequency radio-wave has been widely used in many fields besides ophthalmology [14, 15]. In the present study, patients showed significant improvement in Schirmer test after the procedure (4.04 ± 3.34). Such improvement was not far behind other findings with more invasive techniques [11, 12]. In addition, subjective symptom score of our patients using a self-questionnaire before and after the procedure showed significant improvement.

**Fig. 2:**
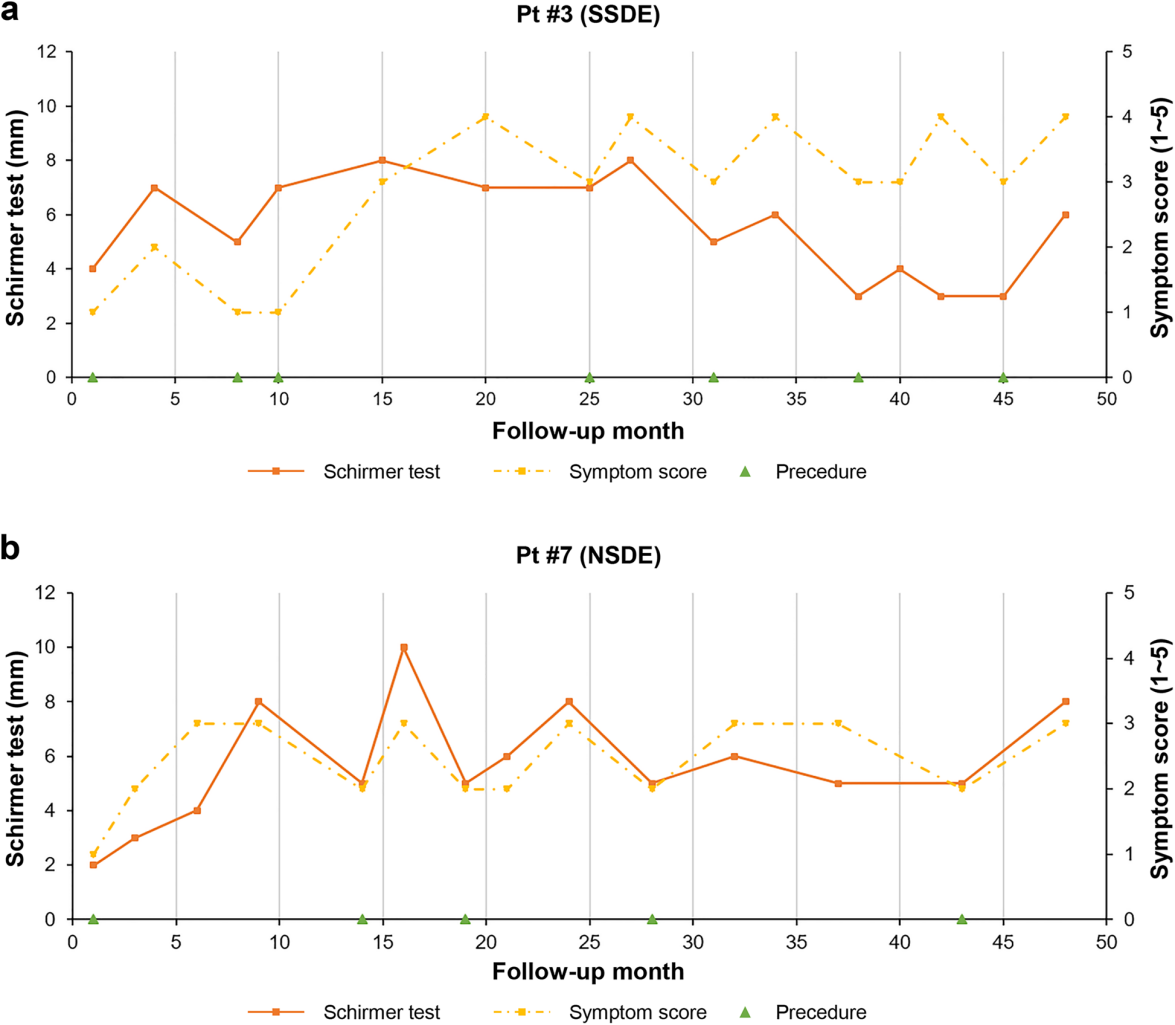
Representative patient case with Sjögren’s syndrome (**a**) and without Sjögren’s syndrome (**b**) who underwent the procedure the most (7 times, 4 times in order). Symptom scores showed improvement as Schirmer test value also showed increase coincidentally

Of 40 patients who participated in this study, 16 patients underwent the procedure more than twice (Table 6). The mean interval between the first and second procedures was 8.00 ± 4.86 months. One patient received the procedure up to 7 times within 3 years, with a mean interval of 7.33 months. The advantage of this procedure is that it does not induce extensive punctal occlusion. In addition, it allows repetition depending on patient satisfaction and symptom change by using less invasive and partial occlusion technique. The SSDE group had a significantly higher number of patients who underwent repeated procedures than the NSDE group. Figure 2 shows changes in the Schirmer test and symptom score for the procedure performed during the follow-up period of patients from each group who underwent the procedure the most during the study period. The change of interval was significant in one patient from 2 to 15 months. It is thought that dry eye syndrome by its nature has high variability in subjective symptoms and anatomical difference in the lacrimal drainage system. Also, there was a lack of standardization of the procedure due to insufficient visualization of the puntal passage during the procedure. However, most patients showed improvement in ocular surface indices after the procedure. They also showed improvement in subjective symptoms. Patients revisited for operation when symptoms relapsed due to recanalization.

**Table 6:** Distribution of patients who have undergone the procedure more than twice

Patients	Age	SS	Total no. of repetition	Mean interval (Mo)
#1	37	+	4	11.67
#2	54	-	2	7
#3	53	+	7	7.33
#4	39	+	3	19
#5	59	-	2	10.33
#6	38	+	2	13
#7	63	-	5	9
#8	61	+	4	7
#9	72	+	2	8
#10	58	-	4	8
#11	55	+	2	10
#12	60	-	3	6
#13	79	-	3	5.5
#14	64	+	2	22
#15	87	+	3	11.5
#16	68	-	2	12

*SS* Sjögren’s syndrome

While this procedure is a simple technique with low risk, it is likely to be re-opened compared to invasive procedures previously introduced in other studies. Additionally, the degree of treatment should be adjusted according to clinical judgment of a surgeon because the therapeutic effect may vary due to individual differences including punctal diameter and canalicular anatomy.

This was a retrospective chart review study and has several limitations. We were unable to analyze the patient's symptom scores in more detail because we did not routinely perform the OSDI in an outpatient setting. In addition, there were limitations in statistical analysis due to missing data and small number of cases. Further studies with prospective design should be performed on a larger number of patients with control group. Therapeutic effects depending on the number of operations on puncta in various kinds of diseases should also be analyzed in further studies.

This technique is relatively less invasive than the conventional punctal occlusion technique. It is a patient-individualized technique that can be easily performed in an outpatient clinic. It is effective without showing any significant side effects in repetitive treatment. Therefore, it can be considered as one of management options for ADDE patients refractory to medical treatment.

## Supplementary Information

**Additional file 1.** Punctal occlusion using high-frequency radiowave. Video clip of the procedure. Insulated fine-needle tip is inserted through canalicular punctum, emitting high-frequency radiowave for 3-4 seconds. Coagulation is repeated for few times per punctum. Punctal opening is relatively spared from coagulation.

## Data Availability

The datasets used and/or analyzed during the current study are available from the corresponding author on reasonable request.

## Abbreviations

TBUT: Tear break-up time
EDE: Evaporative dry eye
ADDE: Aqueous deficient dry eye
SS: Sjögren’s syndrome
DED: Dry eye disease
SSDE: Sjögren's syndrome dry eye
SPSS: Statistical Package for the Social Science
NSDE: Non-Sjögren’s syndrome dry eye
GVHD: Graft-versus-host reaction or disease
OSDI: Ocular Surface Disease Index
